# Rural–Urban Differences in Objective and Subjective Measures of Physical Activity: Findings From the National Health and Nutrition Examination Survey (NHANES) 2003–2006

**DOI:** 10.5888/pcd11.140189

**Published:** 2014-08-21

**Authors:** Jessie X. Fan, Ming Wen, Lori Kowaleski-Jones

**Affiliations:** Author Affiliations: Ming Wen, Lori Kowaleski-Jones, University of Utah, Salt Lake City, Utah.

## Abstract

**Introduction:**

Lower levels of physical activity among rural relative to urban residents have been suggested as an important contributor to rural–urban health disparity; however, empirical evidence is sparse.

**Methods:**

We examined rural–urban differences in 4 objective physical activity measures (2 intensity thresholds by 2 bout lengths) and 4 subjective measures (total, leisure, household, and transportation) in a nationally representative sample of participants in the National Health and Nutrition Examination Survey (NHANES) 2003–2006. The sample comprised 5,056 adults aged 20 to 75 years. Rural-Urban Commuting Area (RUCA) codes were matched with NHANES subjects to identify urban status and 2 types of rural status. Rural–urban and within–rural differences in physical activity were estimated without and with controls for demographic and socioeconomic variables.

**Results:**

Rural residents were less active than urban residents in high-intensity long bout (2,020 counts per minute threshold and 10 miniutes or longer bout length) accelerometer-measured physical activity (42.5 ± 6.2 min/wk vs 55.9 ± 2.8 min/wk) but the difference disappeared with a lower-intensity threshold (760 counts per minute). Rural residents reported more total physical activity than urban residents (438.3 ± 35.3min/wk vs 371.2 ± 12.5 min/wk), with differences primarily attributable to household physical activity. Within rural areas, micropolitan residents were less active than residents in smaller rural areas. Controlling for other variables reduced the size of the differences.

**Conclusion:**

The direction and significance of rural–urban difference in physical activity varied by the method of physical activity measurement, likely related to rural residents spending more time in low-intensity household physical activity but less time in high-intensity physical activity. Micropolitan residents were substantially less active than residents in smaller rural areas, indicating that physical activity did not vary unidirectionally with degree of urbanization.

## Introduction

There is consistent evidence that the rates of overweight and obesity, coronary heart disease, hypertension, stroke, cancer, and diabetes are significantly higher in rural areas than in urban areas in the United States (1–7). Lower levels of physical activity among rural residents relative to urban residents has been suggested as an important contributor to this rural–urban health disparity (1,8–10). However, although some studies found rural residents to be less physically active than urban residents (10–13), others found no rural–urban difference in proportions of residents meeting physical activity recommendations (7). Differences in data sources and measurements have been cited as reasons for the wide range of physical activity estimates among US adults (14). More importantly, studies on rural–urban differences in physical activity have relied on self-reported data, which yield substantially different physical activity estimates than objectively measured estimates (15). In this study we used objective and subjective measures to examine rural–urban differences in physical activity by a nationally representative sample from the 2003–2006 National Health and Nutrition Examination Survey (NHANES). To understand the objective and subjective estimates discrepancy in general and rural–urban differences in particular, we used multiple measures of objective physical activity and multiple categories of subjective physical activity. In addition, we tested the robustness of the potential association between physical activity and degree of urbanization by controlling for demographic and socioeconomic differences.

## Methods

### Sample

NHANES is a program of studies designed to assess the health and nutritional status of adults and children in the United States (16). Since 1999, the survey has examined a nationally representative sample of about 5,000 people each year. NHANES uses a complex, multistage, probability sampling design to select participants who are representative of the civilian, noninstitutionalized US population. The NHANES survey is unique in that it combines interviews and physical examinations. In NHANES 2003–2006, both subjective and objective physical activity data were collected. For objective physical activity data, NHANES participants aged 6 years or older who could walk received accelerometers (Actigraph 7164, LLC, Ft. Walton Beach, Florida) to wear at home for 7 consecutive days. Subjective physical activity data were collected during the interview, which included many questions related to daily activities and specific leisure-time activities. For our study, we focused on adults aged 20 through 75 years who had valid accelerometer data (defined later) and who were not pregnant at the time of the survey. This age range was chosen because studies have shown that adult physical activity does not significantly decline until around age 75 years (17). Using this wide age range allowed us to maximize our rural sample size for a more detailed analysis of rural–urban differences. It also facilitated comparisons of our results with other rural–urban studies that used the NHANES data with the same age range (7,17). Our sample size was 5,056. The study was declared exempt from human subjects research approval by the University of Utah’s institutional review board.

### Dependent variable measures

For *objective physical activity* measures, we used the SAS macro processing NHANES accelerometer data developed by Troiano et al (15). The accelerometer sensor converts movements into electrical signals (counts) that are proportional to the muscular force producing motion. Although Troiano et al used 2,020 counts per minute (CPM) as the moderate-intensity threshold, other studies that used NHANES data used a lower threshold of 760 CPM (18–20). We created measures for both CPM thresholds because Troiano et al speculated that some of the objective–subjective physical activity differences could be attributed to low-intensity physical activity being reported as moderate-to-vigorous (15). Furthermore, in addition to the modified requirement for a 10-minute continuous bout of physical activity in CDC’s *Physical Activity Guidelines for Americans* (21), we computed data on short-bout physical activity that was more than 1 minute but less than the required 10-minutes for both intensity thresholds because misjudging duration could be another contributor to the objective–subjective physical activity disparity. In total there were 4 types of objective physical activity measures (1): high-intensity threshold and long bout length (2,020 CPM or higher and 10 minutes or longer bout length), (2) high-intensity threshold and any bout length (2,020 CPM or higher and any bout length 1 minute or longer), (3) low-intensity threshold and long bout length (760 CPM or higher and 10 minutes or longer bout length), and (4) low-intensity threshold and any bout length (760 CPM or higher and any bout length 1 minute or longer).

For the accelerometer data to be considered valid, participants needed to have worn the accelerometer for 10 or more hours for at least 4 of 7 consecutive days. Nonwear time was defined as 60 consecutive minutes or more of zero activity intensity counts, allowing for 1 to 2 minutes of less than 100 CPM. Wear time per day was defined as 24 hours minus nonwear time (22). Weekly physical activity minutes were computed for each of the 4 measures by multiplying average daily physical activity minutes by 7.

For *subjective physical activity*, the NHANES asked duration and frequency questions regarding leisure activity, household activity, and transportation activity. For leisure activity, NHANES collected data on 48 types of moderate-to-vigorous activities during the previous 30 days, with detailed information on number of times and average duration of activity in minutes. For the purpose of this study, leisure activities were summed together to form one leisure physical activity measure. For household activity, NHANES respondents were asked if and for how long they did any home tasks during the previous 30 days that required moderate or greater physical effort. For transportation activity, NHANES respondents were asked if and for how long during the previous 30 days they had walked or bicycled as part of getting to and from work or school or to do errands. Minutes in these 3 categories were added to form a total physical activity measure. All subjective physical activity measures were converted to average minutes per week.

### Independent variable measures

The definition of “rural” America varies considerably from one study to the next. Most researchers define rural at the county level or metropolitan level as nonmetropolitan areas with a 50,000 population threshold (7,10,23). The US Department of Health and Human Services uses the Rural-Urban Commuting Area (RUCA) codes to determine rurality, a measure developed by the US Department of Agriculture to capture tract-level rurality (24). For this study we used RUCA codes to define rural as all nonmetropolitan census tracts. We further divided rural tracts into nonmicropolitan tracts and micropolitan tracts to explore within-rural differences. The primary 10-category RUCA codes were collapsed into 3 categories as follows (1): nonmicropolitan rural (RUCA = 7–10, area population size ≤ 9,999) (2), micropolitan rural (RUCA = 4–6, area population size 10,000–49,999), and (3) metropolitan/urban (RUCA = 1–3, area population size ≥50,000).The RUCA codes were geo-linked to NHANES by staff at the Centers for Disease Control and Prevention (CDC) Research Data Center. Among the 5,056 respondents in our sample, 624 resided in nonmicropolitan rural tracts, 604 in micropolitan rural tracts, and 3,828 in urban tracts.

*Control variables* included age, sex, self-reported race/ethnicity (non-Hispanic white as the reference group, non-Hispanic blacks, Hispanics, and other race/ethnicities), US-born, education (less than high school, high school or some college as the reference group, and college or more), income-to-poverty ratio, full-time employment, homeowner, marital status, and self-reported poor health. In addition, a variable indicating whether the data were collected between April and October was used as a control because people tend to be more active in summer months than winter months.

Analyses were conducted in 2014 with SAS 9.2 (SAS Institute, Inc) by using procedures that corrected for the complex sampling design of NHANES, as recommended by CDC (25). Sample weights were adjusted for combining NHANES 2003–2004 and NHANES 2005–2006 and for 4 days of valid accelerometer wear, following the methods of Troiano et al (15). We performed domain analyses correcting for sampling design to obtain physical activity estimates, first for urban and rural tracts and then for nonmicropolitan rural tracts and micropolitan rural tracts separately. Wald χ^2^ tests were used to compare physical activity and control variables by rurality. To adjust for demographic and socioeconomic variations between rural and urban tracts, multiple regressions were conducted with controls for variables other than rural or urban. Because significant differences in physical activity were found between those living in nonmicropolitan rural tracts and micropolitan rural tracts, instead of using one combined indicator for rurality, we entered separately 2 dummy variables indicating nonmicropolitan rural status and micropolitan rural status into the regression models. This finer level of detail than that used in previous studies allowed us to detect within-rural differences largely absent from the physical activity literature.

## Results

In the weighted sample, 27.0% (standard deviation [SD] 4.7%) were rural residents: 13.2% (SD 4.0%) resided in nonmicropolitan rural tracts and 13.8% (±3.6%) in micropolitan rural tracts. The other 73.0% (SD 4.7%) resided in urban tracts. Compared with urban residents, rural residents were older; more likely to be white, US-born, married, and homeowners; and less likely to be college graduates. They also had lower income-to-poverty ratios. Micropolitan rural residents differed from nonmicropolitan rural residents: they were more likely to be female, more likely to be black, more likely to be high school graduates, less likely to have less than a high school education, less likely to be married, and less likely to be homeowners. Micropolitan rural residents had lower income-to-poverty ratios than nonmicropolitan rural residents (Table 1).

**Table 1:** Weighted Descriptive Statistics on Physical Activity and Other Variables for Residents (N = 5,056) by Rural–Urban Status: National Health and Nutrition Examination Survey 2003–2006

Variables	Urban (n = 3,828)	All Rural (n = 1,228)	Nonmicropolitan Rural (n = 624)	Micropolitan Rural (n = 604)

	Mean (SD)	Mean (SD)	Mean (SD)	Mean (SD)
Objective physical activity measures (min/wk)				
Physical activity in ≥10 min bouts ≥2,020 CPM	55.9 (2.8)	42.5 (6.2)^a^	52.6 (8.0)	32.9 (4.6)^a^
Physical activity in any bout length ≥2,020 CPM	188.3 (4.3)	162.4 (11.5)^a^	179.9 (18.9)	145.7 (12.0)^a^
Physical activity in ≥10 min bouts ≥760 CPM	314.4 (8.2)	324.8 (26.7)	365.8 (47.1)	285.6 (25.0)
Physical activity in any bout length ≥760 CPM	873.3 (13.3)	895.4 (40.5)	951.0 (75.8)	842.2 (43.1)
Subjective physical activity measures (min/wk)				
Total physical activity	371.2 (12.5)	438.3 (35.3)^a^	495.9 (49.6)^b^	383.2 (23.3)
Leisure physical activity	206.3 (6.3)	206.9 (13.5)	224.3 (25.7)	190.3 (9.7)
Household physical activity	124.4 (8.8)	201.6 (26.3^b^	236.5 (32.5)^a^	168.2 (22.0)^c^
Transportation physical activity	40.5 (4.6)	29.8 (4.9)	35.2 (9.2)	24.7 (4.7)^b^
Demographic, socioeconomic and other variables^d^				
Age, y	43.4 (0.4)	46.4 (0.8)^a^	46.6 (0.8)^a^	46.2 (1.1)^b^
Male	49.7 (0.9)	48.8 (1.3)	50.8 (2.0)	47.0 (1.3)^b^
Non-Hispanic white	66.6 (2.5)	84.3 (4.1)^a^	86.8 (7.2)^c^	81.9 (2.2)^a^
Non-Hispanic black	13.4 (1.7)	6.0 (2.0)^b^	2.3 (1.2)^a^	9.6 (3.5)
Hispanics	13.8 (1.3)	6.2 (3.2)	8.2 (6.0)^a^	4.2 (2.3)^c^
Other races	6.1 (0.8)	3.5 (1.0)^c^	2.7 (1.1)^c^	4.3 (1.3)
US-born	81.1 (1.7)	93.7 (2.1)^a^	93.5 (2.5)^a^	94.0 (3.0)^b^
Less than high school	13.3 (1.0)	15.7 (1.9)	18.1 (1.3)^a^	13.5 (3.5)
High school graduate/some college	55.0 (1.9)	68.0 (2.2)^a^	65.4 (2.5)^a^	70.5 (4.1)^a^
College education or more	31.6 (2.0)	16.3 (1.1)^a^	16.5 (1.6)^a^	16.0 (1.6)^a^
Income-to-poverty (ratio)	3.3 (0.1)	2.9 (0.1)^a^	2.9 (0.1)^a^	2.8 (0.1)^a^
Employed full-time	58.2 (1.3)	56.1 (1.9)	55.1 (3.7)	57.0 (2.8)
Homeowner	68.8 (2.1)	78.2 (3.3)^b^	81.3 (5.2)^c^	75.2 (2.0)^b^
Married/cohabitating	66.6 (1.5)	72.8 (2.0)^b^	75.6 (3.0)^b^	70.1 (2.3)
Poor health	19.3 (0.8)	17.0 (1.5)	18.7 (0.7)	15.8 (1.7)
Data collected April–October	55.9 (5.4)	70.9 (9.2)^a^	70.2 (13.0)	71.6 (14.5)

Abbreviations: SD, standard deviation; CPM, count per minute.

a *P* < .01. Reference category for comparison is urban residents.

b *P* < .05. Reference category for comparison is urban residents.

c *P* < .10. Reference category for comparison is urban residents.

d Values are expressed in percentages unless otherwise indicated.

By using high-intensity threshold (≥2,020 CPM) long-bout (≥10 min) physical activity as the measure, we found that rural residents were less active than urban residents (42.5 ± 6.2 min/wk vs 55.9 ± 2.8 min/wk). However, this rural–urban difference was driven by low activity levels of micropolitan rural residents (32.9 ±4.6 min/wk) compared with nonmicropolitan rural residents (52.6 ± 8.0 min/wk). The same pattern held for high-intensity threshold activity with any bout length. Lowering the intensity threshold to 760 CPM increased physical activity time for rural residents more than for urban residents, resulting in no significant difference between urban residents and either group of rural residents.

Analyses of subjective measures tell a different story. Compared with urban residents, rural residents reported more total physical activity (438.3 ± 35.3min/wk vs 371.2 ± 12.5 min/wk). Among the 3 subcategories of total physical activity, there was no rural–urban difference in leisure activity; however, rural residents reported significantly more minutes of household physical activity than urban residents (201.6 ± 26.3 min/wk vs 124.4 ± 8.8 min/wk). The rural–urban difference in total physical activity was driven more by higher reported activity levels of nonmicropolitan rural residents (495.9 ± 49.6 min/wk) than of micropolitan residents (383.2 ± 23.3 min/wk). Micropolitan residents spent more time in household activity but less time in transportation activity than urban residents, leading to no significant difference in total reported physical activity.

All significant descriptive rural–urban differences remained significant in the multivariate models, although the sizes of the differences were reduced. Compared with urban residents, nonmicropolitan rural residents were not significantly different in all 4 measures of objective physical activity (Table 2) but reported being more active in household activity (80.0 ± 24.9 min/wk) and total activity (107.2 ± 42.1 min/wk) (Table 3). On the other hand, compared with urban residents, micropolitan residents spent less time in high-intensity physical activity (−14.0 ± 6.3 min/wk for long-bout physical activity and −25.6 ± 9.9 min/wk for physical activity with any bout length) but spent about the same amount of time on low-intensity threshold physical activity (Table 2). We found no significant difference in self-reported total, leisure, and household physical activity between micropolitan and urban residents, although micropolitan residents spent less time in transportation physical activity (−15.5 ± 8.2 min/wk). (Table 3).

**Table 2:** Regression Analyses for Objective Physical Activity Measures in Minutes per Week, Comparison of Rural and Urban Residents (N = 5,056): National Health and Nutrition Examination Survey 2003–2006

Variables	Physical Activity, ≥10-Minute Bouts ≥ 2,020 CPM	Physical Activity, Any Bout Length ≥2,020 CPM	Physical Activity, ≥10-Minute Bouts ≥760 CPM	Physical Activity, Any Bout Length ≥760 CPM

	Coefficient (SE)	Coefficient (SE)	Coefficient (SE)	Coefficient (SE)
Rurality				
Urban	1 [Reference]	1 [Reference]	1 [Reference]	1 [Reference]
Nonmicropolitan rural	3.4 (7.3)	2.6 (15.4)	51.2 (39.4)	77.7 (60.2)
Micropolitan rural	−14.0 (6.3)^a^	−25.6 (9.9)^a^	−13.8 (22.7)	−11.7 (31.7)
Age	−0.7 (0.2)^b^	−2.9 (0.2)^b^	−3.9 (0.5)^b^	−8.6 (0.7)^b^
Male	10.4 (5.1)^a^	78.2 (6.7)^b^	145.8 (10.1)^b^	197.0 (13.2)^b^
Race/ethnicity				
Non-Hispanic white	1 [Reference]	1 [Reference]	1 [Reference]	1 [Reference]
Non-Hispanic black	−3.4 (4.1)	−9.4 (6.0)	−13.2 (16.3)	−11.5 (21.9)
Hispanics	−0.1 (7.4)	17.5 (10.1)^c^	70.8 (24.9)^b^	106.6 (32.3)^b^
Other races	−22.5 (6.9)^b^	−47.7 (13.1)^b^	−92.8 (26.4)^b^	−138.4 (37.8)^b^
US-born	−15.9 (5.9)^a^	−29.2 (10.2)^b^	−84.1 (21.1)^b^	−86.1 (27.7)^b^
Education				
High school	1 [Reference]	1 [Reference]	1 [Reference]	1 [Reference]
Less than high school	22.7 (11.2)^c^	29.8 (11.1)^a^	98.2 (21.4)^b^	83.8 (21.4)^b^
College-educated	35.8 (5.5)^b^	24.3 (6.5)^b^	−26.0 (13.8)^c^	−84.9 (16.3)^b^
Income-to-poverty ratio	2.0 (1.9)	3.0 (2.6)	−3.8 (6.2)	−0.4 (8.0)
Employed full-time	−3.0 (4.4)	29.7 (5.8)^b^	59.1 (13.0)^b^	154.0 (21.0)^b^
Homeowner	−2.0 (5.8)	−0.5 (9.1)	23.8 (19.0)	53.3 (22.1)^a^
Married/cohabitating	−1.2 (3.9)	−0.6 (5.2)	9.9 (13.0)	44.1 (18.0)^a^
Poor health	−10.8 (6.0)^c^	−29.5 (5.2)^b^	−61.0 (15.3)^b^	−91.3 (21.6)^b^
Data collected April – October	8.3 (3.4)^a^	19.5 (5.2)^b^	46.7 (11.3)^b^	71.1 (19.8)^b^
Intercept	76.2 (10.0)^b^	259.0 (16.4)^b^	412.5 (39.5)^b^	1051.7 (55.3)^b^

Abbreviations: CPM, counts per minute; SE, standard error.

a *P* < .05.

b *P* < .01.

c *P* < .10.

**Table 3:** Regression Analyses for Subjective Physical Activity Measures in Minutes Per Week, Comparison of Rural and Urban Residents (N = 5,056): National Health and Nutrition Examination Survey 2003–2006

Variables	Total Physical Activity	Leisure Physical Activity	Household Physical Activity	Transportation Physical Activity

	Coefficient (SE)	Coefficient (SE)	Coefficient (SE)	Coefficient (SE)
Rurality				
Urban	1 [Reference]	1 [Reference]	1 [Reference]	1 [Reference]
Nonmicropolitan rural	107.2 (42.1)^a^	29.9 (26.0)	80.0 (24.9)^b^	−2.7 (10.2)
Micropolitan rural	−0.6 (25.9)	−4.0 (10.8)	18.8 (22.7)	−15.5 (8.2)^c^
Age	−1.5 (0.8)^c^	−2.6 (0.5)^b^	1.5 (0.5)^b^	−0.4 (0.3)^c^
Male	111.3 (14.5)^b^	58.0 (9.0)^b^	33.6 (10.5)^b^	19.7 (5.7)^b^
Race/ethnicity				
Non-Hispanic white	1 [Reference]	1 [Reference]	1 [Reference]	1 [Reference]
Non-Hispanic black	12.1 (24.2)	25.4 (13.7)^c^	−29.5 (12.8)^a^	16.3 (8.8)^c^
Hispanics	19.9 (33.3)	35.3 (24.9)	−11.3 (18.5)	−4.1 (10.1)
Other races	34.9 (43.8)	27.4 (26.6)	−7.8 (22.7)	15.2 (10.7)
US-born	75.4 (30.3)^a^	44.6 (16.1)^b^	23.6 (20.3)	7.3 (7.4)
Education				
High school	1 [Reference]	1 [Reference]	1 [Reference]	1 [Reference]
Less than high school	−74.9 (23.7)^b^	−50.7 (14.3)^b^	−34.6 (10.7)^b^	10.4 (12.3)
College-educated	14.0 (19.0)	38.8 (13.8)^b^	−24.8 (9.2)^a^	0.0 (5.7)
Income-to-poverty ratio	5.8 (8.5)	8.7 (5.3)	−0.1 (5.9)	−2.8 (1.8)
Employed full time	−92.6 (23.1)^b^	−48.1 (14.8)^b^	−31.5 (12.9)^a^	−13.0 (7.0)^c^
Homeowner	53.7 (27.4)^c^	16.4 (14.6)	49.6 (11.8)^b^	−12.3 (8.4)
Married/cohabitating	−17.8 (20.9)	−35.5 (14.0)^a^	34.0 (8.7)^b^	−16.3 (6.0)^a^
Poor health	−80.6 (19.7)^b^	−52.2 (10.9)^b^	−15.3 (13.1)	−13.1 (4.4)^b^
Data collected April – October	86.8 (17.6)^b^	36.6 (9.4)^b^	46.8 (9.4)^b^	3.4 (6.4)
Intercept	295.2 (41.1)^b^	240.0 (26.6)^b^	−21.0 (23.9)	76.1 (17.6)^b^

Abbreviations: SE, standard error.

a *P* < .05.

b *P* < .01

c *P* < .10.

We also tested interaction effects between sex and other independent variables. We found that sex was not a significant moderator for rural–urban differences for any of the 4 subjective physical activity measures. However, for objective measures, sex was a significant moderator for high-intensity long-bout activity for nonmicropolitan rural residents. Male nonmicropolitan residents spent significantly less time in high-intensity long-bout activity than their urban counterparts, but this relationship did not hold for females. Sex was also a significant moderator for low-intensity threshold physical activity with any bout length for residents in micropolitan rural tracts. Male micropolitan residents spent significantly more time in low-intensity activity with any bout length than their urban counterparts, but this relationship did not hold for females.

For control variables, the directions of the associations were generally consistent across different measures of physical activity, with a few exceptions. Although older age was associated with less physical activity in general, it was associated with more household activity. Men were more active than women in all activity measures. For objective physical activity, Hispanics spent more time in high-intensity short-bout activity and low-intensity activity than non-Hispanic whites, while other races spent less time in all 4 objective activity categories. For subjective physical activity, the only significant racial/ethnic difference was less time in household physical activity but more time in transportation physical activity for blacks than for whites. Those who were US-born were less physically active than those who were foreign-born according to objective physical activity measures, but the US-born reported more total physical activity time, especially total leisure physical activity. Those with less than a high school education and those with a college degree were generally more active than high school graduates in objective physical activity measures, although high school graduates reported more subjective physical activity than those with less than a high school education. Full-time employment was associated with more short-bout and low-intensity physical activity but less self-reported activity, and being a homeowner and being married was associated with more low-intensity physical activity and more self-reported household physical activity. Poor health was associated with less objective and subjective physical activity measures. People were generally more active in the summer months than winter months.

## Discussion

The most important finding of this study is that the direction and significance of rural–urban differences in physical activity depend on whether objective or subjective measures are used, which intensity threshold of objective measure is used, and which subcategories of subjective physical activity are included. Using objective measures of physical activity with a 2,020 CPM threshold leads to the conclusion that rural residents were less physically active than metropolitan urban residents, whereas using subjective physical activity measures that include household and transportation leads to the conclusion that rural residents were more active than metropolitan urban residents. If the objective physical activity threshold is lowered to 760 CPM, or if only self-reported leisure-time physical activity is used, then there is no rural–urban difference. Our results cannot be compared with results from past studies because of the use of different measures; however, our results do help explain why study findings on rural–urban physical activity differences are so mixed. Indeed, within our data, rural residents were more active, less active, or equally active compared with urban residents depending on the measure used to assess physical activity.

Why would objective and subjective measures of physical activity lead to such different conclusions of rural–urban differences? Studies show that self-reported physical activity levels are substantially higher than objectively measured levels (15,26). One possibility is self-report overestimates, which can result from misclassifying light activity as moderate or from overestimates of activity duration. In our analysis, the main contributor to rural residents reporting more total physical activity than urban residents is that rural residents reported significantly more time spent in household physical activity. It appears that many household physical activity tasks are of lighter intensity than the 2,020 CPM accelerometer threshold counted as moderate-to-vigorous physical activity in our high-intensity objective measures. Indeed, with the low intensity threshold of 760 CPM, the rural–urban differences in objectively measured physical activity no longer exist, indicating that rural residents spent more time in low-intensity activity than urban residents to compensate for less time spent in high-intensity activity. This finding implies that either high-intensity physical activity has health benefits that low-intensity physical activity does not, or other factors such as diet quality or sedentary behavior rather than physical activity are the main contributors of rural–urban health disparity.

An additional finding is that the rural population is not homogenous in terms of physical activity patterns. Within the rural population, micropolitan residents are substantially less active than nonmicropolitan rural residents by almost all measures, objective or subjective. This finding adds information to the literature that disputes the generic notion that physical activity unidirectionally varies with degree of urbanization (10). When sample size allows, future research should look into even more detailed RUCA classifications such as isolated rural versus small townships because the built environment, lifestyle, and social norms may vary substantially with different degrees of rurality in ways we do not currently understand.

Our study has several limitations. Although our sample is nationally representative, our rural sample is not large enough to allow further stratification by sex, race/ethnicity, income, or region, some of which may be important moderators of the relationship between rural and urban physical activity (10,12). Indeed, our tests for interaction effects between sex and rurality showed 1 significant sex effect for nonmicropolitan rural residents and 1 significant sex effect for micropolitan rural residents, indicating that sex could serve as an important moderator for rural–urban differences by some measures of physical activity. In addition, our sample included only respondents with valid accelerometer data. Although we applied weights adjustments to account for age, sex, and racial/ethnic differences in valid accelerometer wear, the possibility still exists that those with valid accelerometer data were systematically different from those without. Also, our measures are not without limitations. Objective accelerometer measures may miss some physical activity that involves upper-body movement only, such as swimming or weightlifting, although the percentage of population involved in these types of activity is small (15). The subjective physical activity measure misses occupational activity, which can be an important component of total activity for some respondents. However, most Americans have occupations with intensity levels too low to meet the moderate-intensity threshold (27).

Despite these limitations, our study has several strengths. First, our use of tract-level RUCA codes as opposed to alternative county-level codes allows us to more accurately identify rural residents. Second, differentiating nonmicropolitan rural from micropolitan rural allows us to uncover physical activity differences within the rural population that have not been investigated before. Third, and most importantly, the NHANES accelerometer data are among the first objective measures of physical activity in a national survey. Combined with subjective measures, we were able to assess multiple measures of subjective and objective physical activity in a nationally representative sample of rural and urban residents to gain insights into rural–urban differences in a more comprehensive way than past studies. To our knowledge, this is the first study comparing rural and urban physical activity by using both objective and subjective measures in a nationally representative sample.
